# Photoluminescence enhancement of quantum dots on Ag nanoneedles

**DOI:** 10.1186/1556-276X-7-438

**Published:** 2012-08-07

**Authors:** Syed Rahin Ahmed, Hee Ryoung Cha, Jung Youn Park, Enoch Y Park, Dongyun Lee, Jaebeom Lee

**Affiliations:** 1 Department of Nano Fusion Technology, College of Nanoscience and Nanotechnology, Pusan National University, Miryang, 627-706, Korea; 2 National Fisheries Research and Development Institute, Busan, 619-705, Korea; 3 Graduate School of Science and Technology, Shizuoka University, 836 Ohya Suruga-ku, Shizuoka, 422-8529, Japan

**Keywords:** Ag nanoneedle film, Nanostructure, Fluorescence, Quantum dots, Plasmon-induced enhancement, Surface roughness

## Abstract

Noble metal nanostructure allows us to tune optical and electrical properties, which has high utility for real-world application. We studied surface plasmon-induced emission of semiconductor quantum dots (QDs) on engineered metallic nanostructures. Highly passive organic ZnS-capped CdSe QDs were spin-coated on poly-(methyl methacrylate)-covered Ag films, which brought QDs near the metallic surface. We obtained the enhanced electromagnetic field and reduced fluorescence lifetimes from CdSe/ZnS QDs due to the strong coupling of emitter wave function with the Ag plasmon resonance. Observed changes include a six-fold increase in the fluorescence intensity and striking reduction in fluorescence lifetimes of CdSe/ZnS QDs on rough Ag nanoneedle compared to the case of smooth surfaces. The advantages of using those nanocomposites are expected for high-efficiency light-emitting diodes, platform fabrication of biological and environmental monitoring, and high-contrast imaging.

## Background

The need for near-field optics has increased tremendously because of the fabrication of metallic nanostructures with tunable surface morphology, allowing unprecedented control over electronic, optical, and mechanical properties [1-8]. Physical and chemical properties of metallic nanostructure are drastically different from those of the bulk solid. This is manifested by a high energy, increased number of defect sites, distinctive electronic state, altered surface crystal structure, and strong absorption at specific wavelength. As a result of these unique features, the metal surface plays a key role in near-field technological applications. In particular, silver film has been frequently employed because of its intrinsic properties such as low contact resistance and low refractive index.

Prominent optical nanostructures include composite structures of semiconducting nanocrystals on these metallic surfaces, which allow us to induce highly efficient emission via excitations in strong coupling regime. In particular, the interfaces are essential in the optical response of semiconductor nanocrystals on metals, where coupling between plasmonic energy and exciton develops [4,9]. This coupling develops new aspects of nanocomposite material systems, inducing many applications that require delicate control of electromagnetic energy in the nanoscale range. For example, Gomez et al. used planer Ag thin film to study the regime of strong light-matter coupling in semiconductor nanocrystals. The demonstration of room-temperature strong coupling involving surface plasmons (SPs) and colloidal quantum dots (QDs) has important practical used of all - optical nonlinear devices, thresholdless laser operation, single-photon optical transistors and spacers (surface plasmon amplification by stimulated emission) that rely on optimizing nanoscale light-matter interaction. Gryczynski et al. studied surface plasmon-coupled emission of semiconductor QDs on a glass slide covered with 50 nm of silver and a 5-nm protective SiO_2_ layer. This might also lead to applications in biological detection, optical coatings, and nanoelectronics [10,11]. However, most research was related to thin metallic film. Very few researches have dealt with the relationship between surface roughness and plasmon-induced photoluminescence (PL) enhancement, even though the roughness of the metallic surface should be a crucial factor for effectively manipulating the interaction between metallic and semiconducting materials in nanoscale devices [12-20].

Therefore, in this paper, metallic Ag nanoneedle films were used for observing luminescence enhancement depending on surface roughness. Structure and roughness of the fabricated sample are predetermined by the structure of the designated template. Fluorescence change was carefully monitored in colloidal CdSe/ZnS core-shell QDs that are resided in the vicinity of two different rough surfaces of Ag nanoneedles, i.e., rough and smooth surface. For the rough surface, vertically aligned Ag nanoneedles (Ag-nNDL) with lengths of a few hundred nanometers and diameters of approximately 50 nm were used as substrates. Meanwhile, a thermally coated Ag substrate was used as the smooth surface. It is probable that the relation between surface roughness and PL enhancement will be a valuable factor to develop further photonic and electronic devices in nanoscale regime.

## Methods

Cadmium oxide (CdO, 99.99%), selenium (99.5%, powder), sulfur (99%, powder), 1-octadecene (ODE, 90%), zinc acetate (99.99%), oleic acid (OA, 90%), trioctylphosphine (TOP, 90%), poly(methyl methacrylate) (PMMA), and chloroform (99.8%) were purchased from Aldrich (Sigma-Aldrich Corporation, St. Louis, MO, USA). All the chemicals were used as received, without further purification.

### Preparation of thermal-coating substrate and Ag-nNDL

A smooth Ag layer was prepared on Si wafer by thermal coating. A thermal evaporator (Thermal Co-evaporator, GEORIMTECH, Daegu, Korea) was utilized at the condition of <1.3 × 10^−3^ Pa and supplying voltage of 0.55 V. Then, it generated 300 nm of thin Ag layer on the substrate. The coated substrate was stored in a desiccator (<30% humidity) before further polymer- and QD-coating process. To produce rough surface, evaporation of silver on the porous template was performed as reported elsewhere [21]. Anodized aluminum oxide (AAO/Al; Nextron Inc., Seoul, Korea) membranes were used as templates; the length and pore size of the AAO membrane were 50 μm and 80 nm, respectively. The prepared AAO membrane was cleaned sequentially in ethanol and deionized water, and then mounted on a thermal evaporator. Evaporation of Ag wire (99.999%) was carried out in a high-vacuum atmosphere (<1.3 × 10^−3^ Pa). The membrane was dissolved with NaOH (2 M) solution at 40°C for 24 h. Then, the residual Ag film was cleaned carefully using ethanol and deionized water. A Si wafer plate was attached to the rear side of the film for easy handling.

### Synthesis of CdSe/ZnS QDs

As with a typical synthetic procedure based on the previous reference [22], 0.05 mmol cadmium oxide and 2 mmol zinc (acetate)_2_ were dissolved in a mixture of 3.5 mL oleic acid (OA) and 8 mL 1-octadecene in a 50-mL round flask. The solution was degassed and purged with N_2_ for 30 min, heated to 180°C to obtain a pale yellow solution, and then cooled to 100°C, at which the reaction solution was degassed for 20 min. Then, the reaction mixture was further heated to 300°C, yielding a clear solution of Cd(OA)_2_ and Zn(OA)_2_. At the elevated temperature of 300°C, 0.1 mmol Se and 1.5 mmol S, both dissolved in 1.5 mL trioctylphosphine, were swiftly injected into the hot solution, and the color of the solution turned light red immediately. Continuous heating at 310°C for 10 min allowed the QDs to emit green color of fluorescence.

### QD coating on the smooth and rough substrates

Before the coating process, the Ag-nNDL film was cleaned carefully by sequential treatment with ethanol and deionized water to remove any organic residues. Then, it was dried gently by filtered N_2_ gas under ambient conditions. Colloidal CdSe/ZnS QDs were first dissolved in chloroform to obtain a dilute solution (approximately 1.4 × 10^−10^ M). To serve as a spacer between the Ag film and the QDs, a 1% solution of PMMA in toluene was spin-coated onto two different Ag films at 4,000 rpm for 40 s and cured at 60°C to create a thin layer. The root mean square of roughness, *R*_RMS_, of the respective films was calculated in at least five different areas of 10 × 10 μm. From the surface morphological images of atomic force microscopy (AFM; Veeco Instruments Inc., Plainview, NY, USA), the *R*_RMS_ of each sample, i.e., smooth and rough substrates, were 1.8 ± 0.12 nm and 39.7 ± 1.08 nm, respectively. The polymer-coating process allows the QDs to be associated with plasmon resonance while preventing luminescence quenching due to charge transfer between the QDs and the Ag surface [23]. Then, the 100-μL QD solution was spin-coated over the PMMA thin film at 4,000 rpm for 40 s at room temperature.

### Optical and microscopic measurements

To measure the absorbance of the QDs on the Ag-nNDLs, the absorbance reflection mode was utilized in UV-visible (UV–vis) spectroscopy (SCINCO S-3100, SCINCO Co., Ltd., Seoul, Korea). The PL enhancements of the samples were measured by a fluorescence spectrophotometer (Model No. F-7000, Hitachi, Tokyo, Japan) when the angle of incident light was 45°. Fluorescence lifetimes (*τ*) of the respective samples were measured at a 380-nm excitation wavelength using a light-emitting diode (LED; PTI Inc., Oakland, CA, USA). Note that this wavelength is similar to the wavelength of the surface plasmonic field of individual Ag-nNDLs, which would enhance the signal-to-noise ratio of the gathered fluorescence from the QDs deposited on the Ag substrate. Possible spurious scattering of light was blocked by installing optical filters and slits that are as narrow as possible. Topographic images of Ag-nNDL surfaces were obtained using AFM (diInnova, Veeco, USA) and scanning electron microscopy (SEM; Hitachi-S4700, Japan).

## Results and discussion

### Topographic observation of Ag-nNDL film

**Figure 1:**
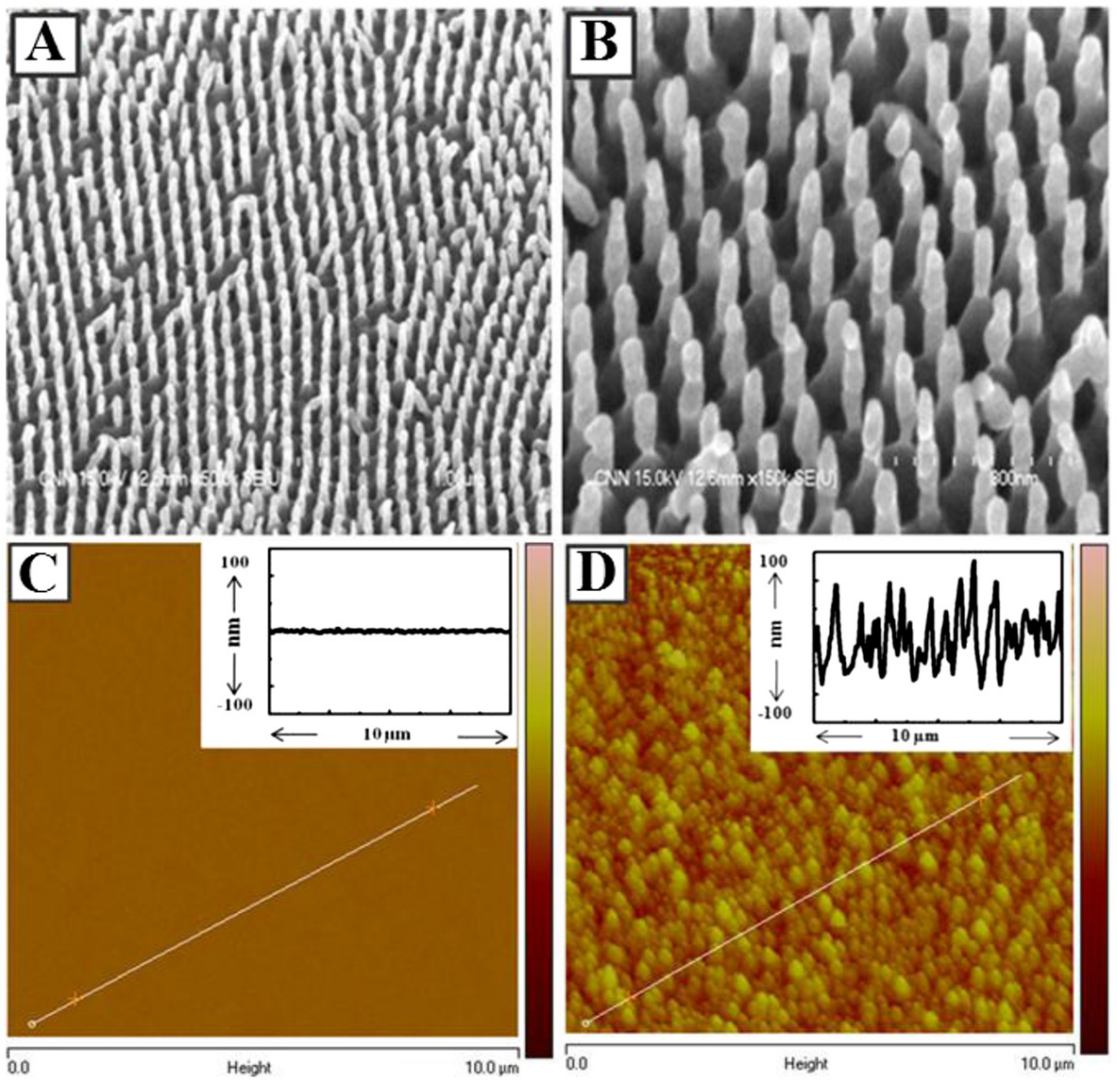
**SEM images of Ag-nNDLs at × 50,000 (A) and × 150,000 (B).** Topographical AFM images of Ag films: **(C)** smooth Ag substrate and **(D)** rough Ag-nNDLs. Insets show the depth profiles along each line.

Figure 1 shows morphologies of the smooth and rough Ag films, i.e., thermal-coated and nNDL substrate. The conical end shapes of the Ag-nNDL were arrayed in an average distance of 350 nm. The shapes and sizes of the needles were relatively regular and ordered in a large area (over a few micrometers). The needle diameter was 50 ± 1.25 nm, which is smaller than the pore size of the AAO. The average length of the Ag-nNDLs was about 197 ± 4.03 nm. The end shape of each nanoneedle was not regular since Ag needles were fabricated while the AAO entrance was being blocked. Normally, the size and shape of the metallic end are dominant factors affecting the plasmonic enhancement of fluorescent materials, particularly in a single-NP-level experiment [24,25]. However, in our experiments, many QDs were coated on the Ag-nNDLs with no order. This reduced the size and shape effects but show collective optical response of total enhancement from many irregularly coated QDs on the metallic surface, truly by *roughness effect*. The *R*_RMS_ was observed from topographic images obtained by AFM tapping mode (Figure 1C, D). The *R*_RMS_ of the smooth surface was about 1.8 ± 0.12 nm, whereas the other *R*_RMS_ was 39.7 ± 1.08 nm. It indicates that the particle size and distribution were relatively uniform for the smooth surfaces. However, the surface topography was quite irregular, and the size distribution was uneven when the Ag-nNDLs were grown. The insets in Figure 1C, D show the depth profiles along the solid lines, respectively. The average height of the smooth Ag surface was approximately 6 nm, while for the rough surface, it was measured at over 100 ± 2.62 nm on average. This was slightly lower than the measured length determined from the SEM image, which might be due to detection allowance of the tapping mode in AFM. 

### QD coating on smooth and rough substrates

**Figure 2:**
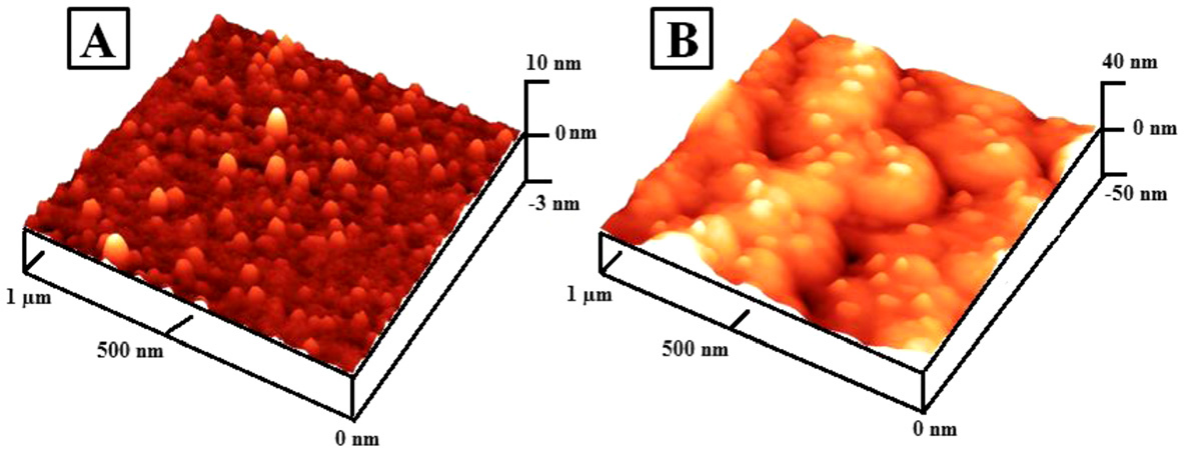
AFM images of smooth (A) and rough (B) substrate after QD coating.

Figure 2 presents the surface morphology of the smooth and rough surfaces after QD-coating processes as described in the ‘Methods’ section. The sequential coating of polymers and QDs showed a well-distributed surface on both Ag surfaces, respectively. In Figure 2A, most QDs were well dispersed on the smooth Ag substrate, which may decrease the quenching of PL by aggregation. In Figure 2B, most QDs were scanned on top of the Ag-nNDL where deep-area scanning by AFM imaging may be limited. It is probable that the aggregation of QDs on the rough surface was also minimized due to a surface area larger than the smooth one.

### Spectroscopic study of QDs on Ag substrates

**Figure 3:**
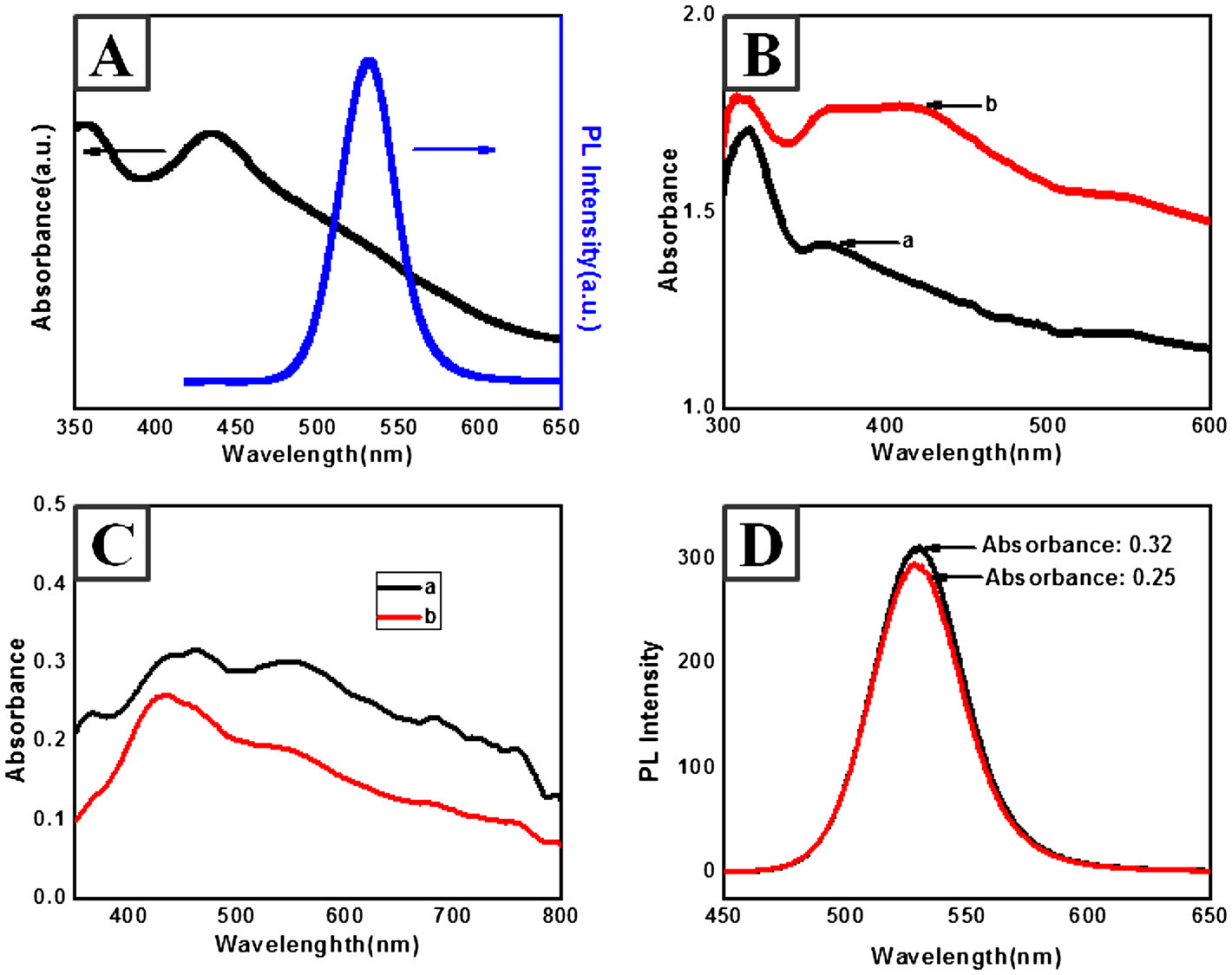
**UV and PL spectra of CdSe/ZnS QDs (A) and UV–vis spectra of Ag-nNDLS (B).** a, smooth Ag nanofilm; b, Ag-nNDL. **(C)** Absorbance spectra of QDs on each film. **(D)** Fluorescence spectra of QDs in chloroform at the same absorbance intensity.

Figure 3A shows typical absorption and PL spectra of the synthesized CdSe/ZnS QDs. The absorption spectrum of QD was shown with the highest peak at 450 nm, and the fluorescence band was very narrow at 100 nm, with a quantum yield (QY) of >20%. The quantum yield was measured by the relative ratio against rhodamine dispersed in ethylene glycol (its QY as 0.95). Figure 3B illustrates the typical UV–vis spectra of the smooth and rough Ag-nNDLs. Absorption spectra of the prepared substrates were measured in the reflection mode. For the smooth substrate (image in Figure 1C), the UV spectrum revealed an absorption maximum (λ_max_) at 360 nm, which corresponded to the plasmon band of Ag nanomaterials because the thermally coated layer was approximately 300 nm. For the rough substrate, λ_max_ showed a bathochromic shift from 360 to 420 nm with a plateau region. The shift would be crucial to affect PL enhancement of the deposited QDs by plasmonic coupling.

**Figure 4:**
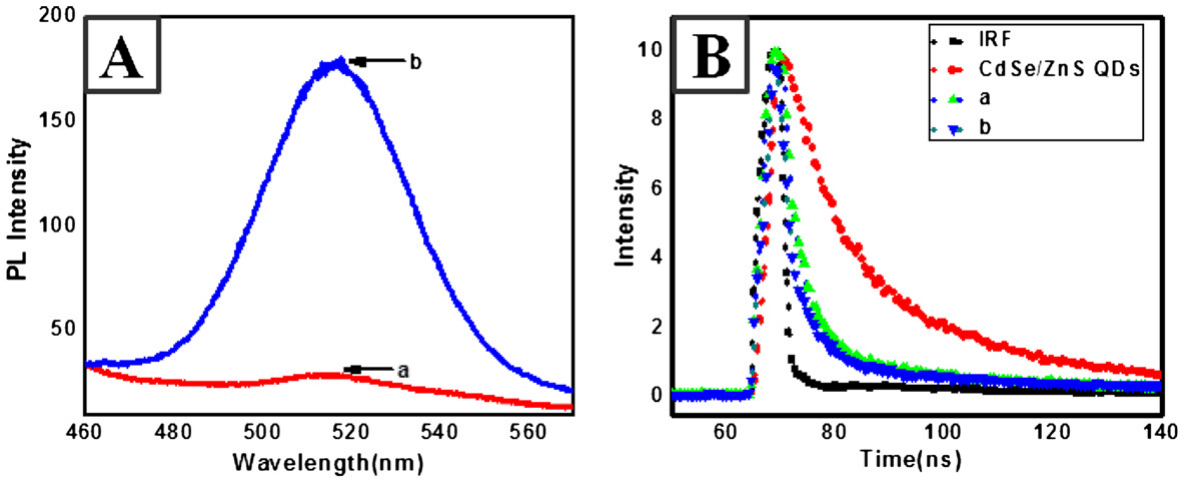
**PL spectra for CdSe/ZnS nanocrystals on silver surfaces (A).** a, smooth surface; b, rough surface. **(B)** Time profile of PL signal for CdSe/ZnS nanocrystals on Ag surfaces.

Since the roughness of the produced films was different, special care was required in the QD-coating process in order to deposit the same amount of QDs on each substrate. In the coating process, the absorbances of QDs on the surface of the respective substrates were monitored to maintain similar intensities since absorbance corresponds to PL intensity. The absorbance was monitored by UV–vis spectroscopy after QD deposition on the surface of each substrate (Figure 3C). The absorbance difference between two substrates after QD deposition was about 0.07 at the wavelength of 380 nm where the QD was excited. Fluorescence intensity of the QD at this absorbance difference was monitored in solvent state. The PL spectra in Figure 3D showed a small difference in the PL intensity between the two substrates, from which it is probable that similar amounts of QDs were deposited on the respective substrates, and these results can be comparable to the striking PL intensity variance of QDs depending on the different roughness of substrates in Figure 4A.

Figure 4A shows the PL spectra obtained for CdSe/ZnS QDs on the two different Ag substrates at the excitation wavelength of 380 nm. A dramatic enhancement in the PL intensity from the QDs on the rough surface of Ag-nNDLs was observed. About six-fold enhancement was observed compared with the PL intensity on the smooth surface. It is probable that the huge PL change stems from the different surfaces, i.e., the roughness difference or the aggregation control of the deposited QDs. First, such aggregation on the substrate may induce a decrease in the PL intensity. In the evaporation step after coating, the distance between the QDs decreased, and dipole-dipole attraction was more pronounced than charge-charge repulsion. Furthermore, the continuous surface tension during the evaporation forces the congregation of QDs into localized areas. However, on the rough surface, aggregation can be dramatically avoided because of the extremely large surface area. The physical barrier of the Ag-nNDLs decreases the surface tension energy, which may induce fast solvent evaporation after coating. These factors should limit the movement of QDs on the surface, with respect to gathering in a localized area. However, the avoidance of aggregation would not be the main factor for PL enhancement because most of the aggregation was intentionally avoided during the coating process by adjusting the concentration of deposited QDs. Furthermore, note that the detailed imaging of AFM proved less aggregation in Figure 2. When similar amounts of QDs were deposited on the respective substrates, the intensity difference between them was very small, which may strongly support the vital role of the plasmonic rough surface in enhancing fluorescence emission Figure 3D.

PL decay was measured using a 380-nm excitation source at room temperature. Figure 4B shows the PL lifetime profiles of the QDs on the two different Ag surfaces. Clearly, the PL decay on the rough surface was faster than that on the smooth surface. The decay rate of QDs was 9.303 ns, and the decay rates of the CdSe/ZnS nanocrystals on the smooth and rough surfaces were about 2.156, 1.445 ns, respectively, which are much shorter than that of QD. Even the rough surface showed shorter decay rate than the smooth Ag surfaces. The standard deviation of decay was <5% after measurement of different samples ten times. It is probable that the shorter PL lifetimes on the rough surfaces are governed by the energy transfer rate that is faster than that in the case of the smooth surfaces.

Interactions between excitons and plasmons occur when metal and semiconductor nanostructures are in close proximity. The noble metallic surface usually displays a plasmon resonance arising from the collective oscillation of migrated electrons. The radiating energy from the QDs is dramatically altered through coupling with the metal plasmon resonance, which causes a change in the emission properties. However, one often discerns two opposite cases of weak and strong coupling. In the weak coupling regime, wave functions and electromagnetic modes of excitons and plasmons are relatively unperturbed, and exciton-plasmon interactions are often described by the coupling of the exciton dipole with the electromagnetic field of the SP. This model has been used to explain the original experiments of an emission dipole in the proximity of a plane metal surface where plasmonic scattering is insufficient and energy dissipates thermally, resulting in minimal fluorescence enhancement. Meanwhile, the strong coupling regime is considered when resonant exciton-plasmon interactions modify exciton wave functions and SP modes and lead to changes of exciton and SP resonance energies that are larger than their natural line widths. Typically, an evanescent field created by total internal reflection at the interface is used to realize supercritical light propagation (forbidden light) in random media. According to near-field optics, both metallic surfaces and nanocrystals act as tiny particles. In this regime, the excitation energy is shared and oscillates between the plasmonic and excitonic systems (Rabi oscillations), and typical anticrossing and splitting of energy levels at the resonance frequency are observed. These energy resonances adsorbed by the QDs result in the plasmon interaction, which could amend the energy gap between the exciton hole and the trapped hole, and thus eliminate the hole-trapping process, omit unwanted nonradiative energy transfer, and lead to enhanced fluorescence emission. The strong coupling regime is observed in heterostructures due to the efficient radiative scattering without any loss of energy, which would lead to interactions between quantum-confined electronic states in semiconductor nanostructures and dielectric-confined electromagnetic modes in the metal counterparts. Such exciton-plasmon interactions allow the design of absorption and emission properties, control of nanoscale energy transfer processes, and creation of new excitations in the strong coupling regime [26,27].

The fluorescence lifetime is also sensitive to the local environment of the QDs. The spontaneous emission decay rates of the optically excited QDs are accelerated by the locally enhanced electromagnetic field in the vicinity of the metallic structure. The possibility of energy transfer between the excitonic and the plasmonic systems depends on the local environment. The surface plasmon coupling technique has the potential to enhance the spontaneous emission rate. For a smooth surface, energy transfer can be considered unidirectional due to the weak exciton-plasmon coupling. The energy is transferred to the metal or semiconductor subsystem that either spatially removes the energy from the interaction zone or rapidly relaxes the energy to lower energy levels, thereby omitting resonant back transfer. In the strong coupling regime, i.e., the rough surface, the energy is stored in both (metallic surface and QDs) and oscillates back and forth between excitons and surface plasmons (Rabi oscillations). The decoherence time of surface plasmons in this case is on the femtosecond time scale and is often shorter than the time required for Rabi oscillations. The scattering of light due to surface roughness is one of the effects that can cause substantial losses and can significantly reduce the SP decoherence time, which is considered to increase radiative decay rates and shorten the fluorescence lifetime.

## Conclusions

In this paper, we present a SP-enhanced QD emission due to scattering on rough Ag nanoneedle films. Our results strongly suggest that the locally enhanced electromagnetic field due to the scattering of SP energy on a rough surface results in a more effective QD excitation, as compared to the case of QDs on smooth surfaces. The strong metal-QD interaction due to the heterogeneous surface topography of the silver nanostructure also dramatically shortens the lifetime of the non-emissive state. The methodology and observations reported here could be relevant for the design and construction of high-efficiency light-emitting diodes, platform fabrication of biological and environmental monitoring, and high-contrast imaging.

## Competing interests

The authors declare that they have no competing interests.

## Authors’ contributions

SRA carried out the QD synthesis and spectroscopic studies, and HRC prepared the Ag nanoneedle films and microscopic studies. Both authors drafted the manuscript. JYP and EYP participated in the design of the study. DL and JL conceived of the study and participated in its design and coordination. All authors read and approved the final manuscript.
